# Experimental and Theoretical Studies on Corrosion Inhibition of Niobium and Tantalum Surfaces by Carboxylated Graphene Oxide

**DOI:** 10.3390/ma11060893

**Published:** 2018-05-25

**Authors:** Valbonë Mehmeti, Fetah I. Podvorica

**Affiliations:** FNMS, Department of Chemistry, University of Prishtina, Prishtina 10000, Kosovo; valbonamehmeti1@gmail.com

**Keywords:** niobium, tantalum, carboxylated graphene oxide, nanomaterials, corrosion inhibition, molecular dynamics, Monte Carlo calculation

## Abstract

The corrosion of two different metals, niobium and tantalum, in aqueous sulfuric acid solution has been studied in the presence and absence of carboxylated graphene oxide. Potentiodynamic measurements indicate that this nanomaterial inhibits corrosion due to its adsorption on the metal surfaces. The adsorbed layer of carboxylated graphene hinders two electrochemical reactions: the oxidation of the metal and the transport of metal ions from the metal to the solution but also hydrogen evolution reaction by acting as a protective barrier. The adsorption behavior at the molecular level of the carboxylated graphene oxide with respect to Nb, NbO, Ta, and TaO (111) surfaces is also investigated using Molecular Dynamic and Monte Carlo calculations.

## 1. Introduction

In general, metals are prone to oxidation when exposed to different aggressive solutions in the course of industrial cleaning or other diverse treatments (acid pickling, descaling and cleaning). Although the use of paints and polymers offers a simple approach to decrease the effect of corroding species on the surface, the major drawbacks of this method are the weak interactions among the metal surface and the coating layer so the physical organic barrier is deteriorated after a short time of exposure [1]. The widespread method of the protection of metals against corrosion remains the use of inhibitors molecules [2,3,4]. These moieties when added at relatively small concentrations in the corrosive media where the metal is exposed can interact with its surface, forming an organic coating and therefore decreasing the corrosion rate. Other approaches for corrosion inhibition rely on the use of surface modification strategies through the formation SAM’s (Self Assembled Monolayers) using phosphonic acids [5,6], silanes, aryl diazonium salts [7], etc. Graphene is considered as one of the most important classes of modern materials-nanomaterials [8], which are extensively explored for use in different fields of science from drug delivery [9] to photovoltaics [10]; however, graphene remains almost unexplored towards applications aimed at the corrosion inhibition of materials. Only limited scientific research that uses graphene nanomaterials is devoted to corrosion studies [11,12,13], and to our knowledge there is no reported study on the use of carboxylated graphene oxide (GO-COOH) as a corrosion inhibitor. The production of nanomaterials is steadily increasing [14,15] using low-cost, abundant natural materials [16]; therefore, the future use of nanomaterials in corrosion protection is promising.

Quantum chemical methods are widely employed for the study of the physico-chemical properties of the inhibitors in order to understand at the molecular level their interaction with the metal surface [1,4,17,18]. Density Functional Theory (DFT), as a powerful technique for performing calculations on many bodied systems, permits the correlation between the adsorption ability of the inhibitor molecule on the metal surface and its electron donating tendency that is expressed by the value of E_HOMO_. Higher values for this orbital suggest an increasing donor-acceptor interaction between the inhibitor molecule and the vacant d-orbitals of the metal surface atoms [19,20]. DFT calculations also make possible the estimation of other parameters like the dipole moment, electronegativity and ionization potential energy [21]. For example, the inhibitor efficiency increases when the metal surface is covered with higher dipole moment molecules due to the electrostatic interaction between the charged metal surface and charged centers of molecules [22]. Molecular dynamics simulation provides more insight into the corrosion inhibition mechanism and the different interactions at the interface inhibitor-metal surface [23]. However, for larger systems, these methods are computationally costly to employ.

In this work, molecular dynamics [24,25] and Monte Carlo [26] simulation are used to evaluate the interaction between the Nb (111), NbO (111), Ta (111) and TaO (111) and carboxylated graphene. 

## 2. Materials and Methods 

### 2.1. Experimental Section

Carboxyl Graphene (Dispersion, 5 mg/mL, ACS Material, Pasadena, CA, USA) dissolved in aqueous solution of H_2_SO_4_ at c = 0.1 mol/dm^3^ was used at a concentration of 2.5 × 10^−2^ mg/mL. The GO-COOH was well dispersed. For the electrochemical measurements, the electrodes were prepared by embedding a niobium wire (*d* = 1 mm, *l* = 10 mm, (annealed), 99.8% (metals basis), Alfa Aesar^®^, CAS: 7440-03-1, Karlsruhe, Germany) and tantalum wire (*d* = 0.5 mm, *l* = 10 mm, (annealed), 99.95% (metals basis), Alfa Aesar^®^, CAS: 7440-25-7, Karlsruhe, Germany) inside a Teflon^®^ (*d* = 1 cm, *l* = 6 cm, Alfa Aesar^®^, Karlsruhe, Germany) tube with epoxy resin. Prior to use, the electrodes were polished on silicon carbide abrasive paper (medium grain diameter 6.5–15.3 microns), then on a (DP-Nap) cloth with an aluminium oxide (0.3 micron particle size) suspension, and then the electrodes were washed and sonicated in water.

Sulphuric acid 97%, pro analysis, MERCK, UN-No.1830 (Darmstadt, Germany) is used as the electrolyte for the potentiodynamic measurements. Its concentration in whole measurements was 0.1 mol/dm^3^. 

The electrodes were made of commercial niobium (*d* = 1 mm) and tantalum (*d* = 0.5 mm) wires. Prior to electrochemical measurements, metal surfaces were mechanically polished with emery paper, cleaned with distilled water and degreased in ethanol, washed with distilled water and finally dried in air. The electrochemical studies and linear sweep voltammetry (LSV) were performed using an Autolab potentiostat (Metrohm Autolab, Utrecht, Netherland) and employing a three-electrode cell assembly. A saturated calomel electrode (SCE) and platinum electrode were used as reference and auxiliary electrodes, respectively. All solutions were prepared from analytic grade chemicals and bidistilled water. The metal electrodes were allowed to stabilize their open circuit potential (OCP) until the potential stabilization criteria of dE/dt limit 10^−6^ V/s is reached. Potentiodynamic polarization was carried out by scanning the potential ±250 mV from the evaluated OCP using a scan rate of 1 mV s^−1^. Polarization experiments were carried out using a potentiostat from Eco Chemie BV, the Netherlands, Autolab PGSTAT128N (Metrohm, Utrecht, The Netherland), and the obtained data were analyzed with Autolab software (NOVA 2.0.1, Metrohm, Utrecht, Netherland).

### 2.2. Monte Carlo Simulations

**Figure 1:**
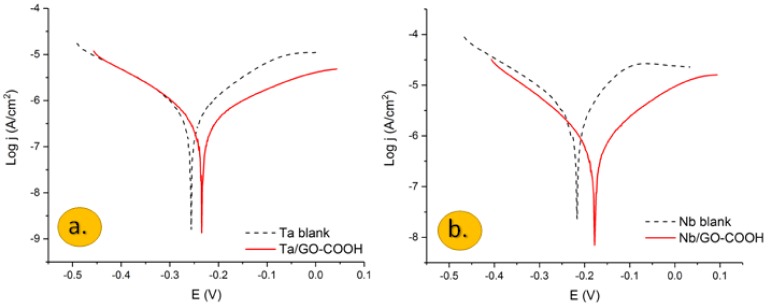
Semilogarithmic polarization plots of: (**a**) Ta and (**b**) Nb electrodes in a 0.1 M H_2_SO_4_ aqueous solution with and without the presence of 100 ppm GO-COOH. Ref. SCE. v = 1 mv/s.

Adsorption Locator module in Materials Studio 7.0 was used to build Nb (111), NbO (111), Ta (111) and TaO (111). The carboxylated graphene model (Figure 1) was built using Avogadro software (version no.1.0.1, Softonic, Barcelona, Spain) and was optimized using Universal force field (energy convergence tolerance of 2 × 10^−5^ kcal/mol, force convergence tolerance of 0.001 kcal/mol/A). The molecular dynamic simulations of the interaction between the GO-COOH studied as inhibitor and the surfaces were carried out in the simulation box with corresponding dimensions: [Nb (111)—28.00 Å × 28.00 Å × 5.47 Å; NbO (111)—28.32 Å × 26.79 Å × 6.31 Å; Ta (111)—28.02 Å × 28.02 Å × 4.67 Å; TaO (111)—31.26 Å × 31.26 Å × 6.63 Å using periodic boundary conditions with a of 20 Å vacuum along the C-axis. The solvent (water) effect was simulated by loading 50 water molecules (geometrically optimized using Universal forcefield) into the simulation box together with the studied molecules (using the same optimization algorithm). The Metropolis Monte Carlo method was used to evaluate the adsorption configurations (Universal, force field) of the interaction between the molecule and the substrates. 

### 2.3. Molecular Dynamic (MD) Simulations 

The MD simulations with the Forcite Module (Materials Studio) were performed using Universal Forcefield. Prior to the use of MD simulations, the surfaces were optimized using the Smart optimization algorithm with the energy convergence criteria of 10^−4^ kcal/mol and force criteria of 5 × 10^−3^ kcal/mol/Å. The atom charges were assigned using the QEq method with the atom-based electrostatic and Van der Waals summation method (truncation: cubic spline, cutoff distance 15.5 Å, and spline width 1 Å). The optimized structure of GO-COOH was added to the optimized slab surface along the perpendicular directions. A “vacuum layer" with a height of 50 Å was placed upon the surface with PBC (periodic boundary conditions). The MD was conducted using an NVT (constant-temperature, constant-volume) canonical ensemble at 298 K. The time step for MD was 1 fs with a total simulation time of 300 ps (3 × 10^5^ steps). The system temperature was maintained using a Berendsen Thermostat (0.1 ps decay constant). For the data analysis, 300 ps of trajectory frames were used.

### 2.4. Adsorption Energy and Radial Distribution Function

The adsorption energy was calculated using the following equation:

E_adsorption_ = E_total_ − (E_surf._ + E_GO-COOH_)/slab area (1)

The radial distribution function (RDF) [22,23,27,28] defines the probability of finding adsorbate molecules at distance r from the surface atoms of the slab models. It was calculated as follows:

g(r) = (n(r))/2πrΔrρ(2)

where r (A) is the given distance from surface atoms from the slab, n(r) is a time-averaged number of atoms in the area of r ± ∆r, ρ is the number density of the system.

## 3. Results and Discussion

### 3.1. Potentiodynamic Polarization

Polarization plots obtained for the Nb and Ta electrodes in a 0.1 M H_2_SO_4_ aqueous solution in the presence and the absence of carboxylated graphene oxide are shown in Figure 1a,b.

Potentiodynamic measurements, in the case of the Ta and Nb bare electrodes, showed a fast increase of the anodic current, after the corrosion potential. In both cases, there is an active dissolution of tantalum and niobium due to the formation of Ta^5+^ and Nb^5+^ ions, respectively, which slow down around −0.05 V.

According to the Pourbaix diagrams for the Ta-H_2_O and Nb-H_2_O [29,30], in aqueous sulphuric acid solution at 25 °C at pH value > 0.5, Ta and Nb are oxidized to Ta_2_O_5_ and Nb_2_O_5_; therefore, the formation of passive oxide layers prevents further oxidation of metals when they are under positive potential [31,32].

The inhibition behavior of the GO-COOH layer is evidenced by the decreased anodic and cathodic current densities.

The kinetic electrochemical corrosion parameters, corrosion current density (Icorr), cathodic and anodic Tafel slopes and corrosion potential (Ecorr), were calculated from the extrapolation of Tafel plots, and the values are presented in Table 1.

Even though the corrosion current density decreases in the presence of GO-COOH, the corrosion potential does not change significantly in the presence of the inhibitor. The anodic Tafel slope is somewhat larger than that of the cathodic one, and the corrosion potential is slightly displaced anodically, showing that this protective barrier acts mainly as an anodic inhibitor. Graphene layers are well known for the creation of adhered coatings on metal surfaces and affect the slope of anodic and cathodic reactions and hinder the corrosion reaction due to the large specific surface area and excellent mechanical properties [11,33]. These layers may hinder the diffusion of water molecules to the electrode surface [33].

The inhibition efficiency (IE) was calculated from the corrosion current density of the metal electrodes in the absence and presence of the inhibitor using the following equation: it reached 70% and 64% for Nb and Ta, respectively.

IE (%) = (I_unhib_) − (I_inh_)/(I_unhib_) × 100 (3)

**Figure 2:**
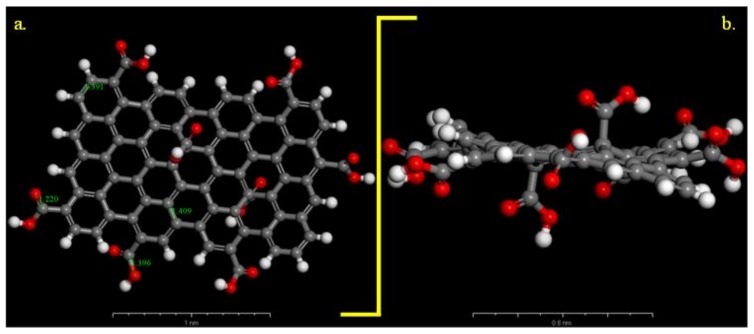
Optimized carboxylated graphene model (Universal forcefield): (**a**) on top view and (**b**) side view. Gray colour: C atoms, white colour: H atoms and red colour: O atoms.

We compared these experimental results with those of the differential simulations. The Universal force field correctly describes the C–C, C–O distances in the GO-COOH structure, Figure 2. The bond lengths for the computed GO-COOH are in good agreement with the generally found experimental bond lengths and are as follows: –C=C– (1.409 Å), –C=O (1.22 Å), C–O(H) (1.39 Å). We have considered that all COOH groups were protonated due to the low pH value of the aqueous solution of 0.1 M H_2_SO_4_ so the inhibitor is a neutral molecule and any influence of protons is assumed to be negligible.

**Figure 3:**
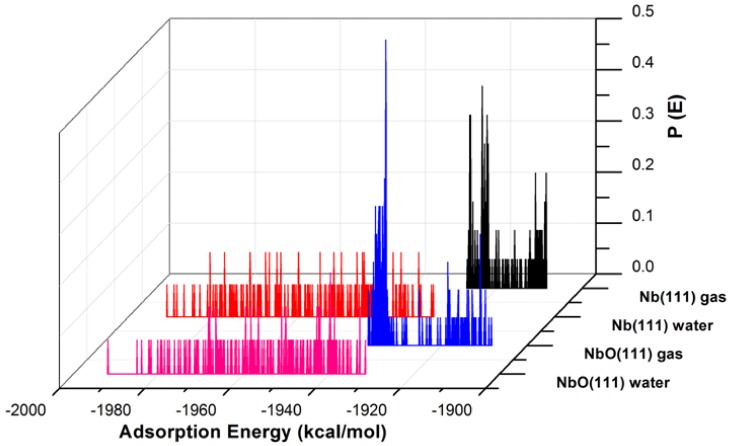
The adsorption energy distribution of the GO-COOH on Nb (111) and NbO (111) surfaces in the presence and absence of water.

**Figure 4:**
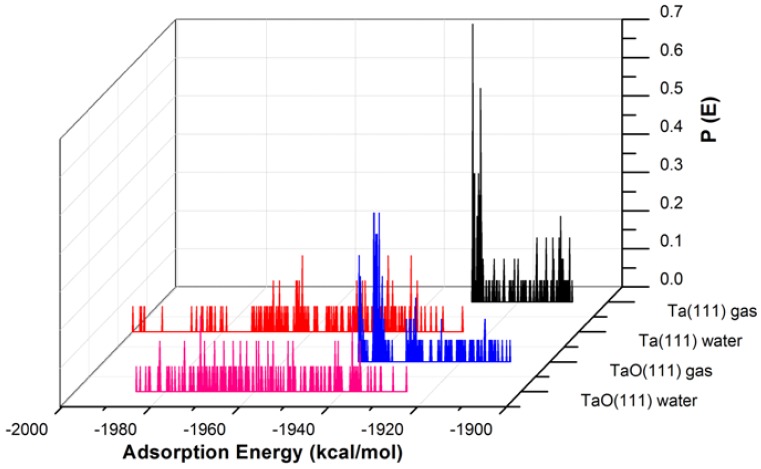
The adsorption energy distribution of the GO-COOH on Ta (111) and TaO (111) surfaces in the presence and absence of water.

The adsorption of GO-COOH evaluated from the Monte Carlo simulations on both surfaces exhibits many adsorptive configurations, see Figure 3 and Figure 4. These configurations arise from different approaching paths and geometries of the GO*x* molecule. 

**Figure 5:**
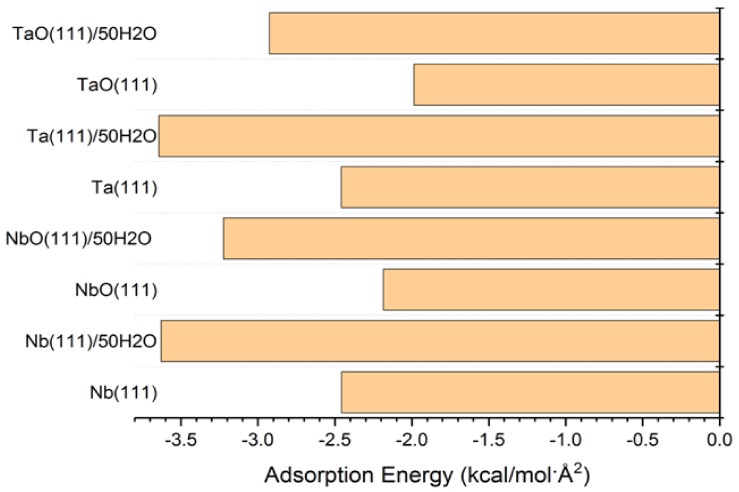
Adsorption energies calculated by the Monte Carlo simulation of GO-COOH on Nb (111), NbO (111), Ta (111) and TaO (111) surface, in the presence and absence of water.

The adsorption energies presented from the MC simulations are summarized in Figure 5. This figure shows the distribution of potential energy as a function of adsorption energy, and the minimum adsorption is located on the left side of the axis. The adsorption energy for Nb (111) and Ta (111) surfaces divided per surface area of the used slab models is comparable for both the gas phase and in the presence of water molecules. The presence of water, as expected, favors the adsorption of GO-COOH, as it decreases the adsorption energy (the energy gain is around −1.8 kcal/mol·Å2). 

**Figure 6:**
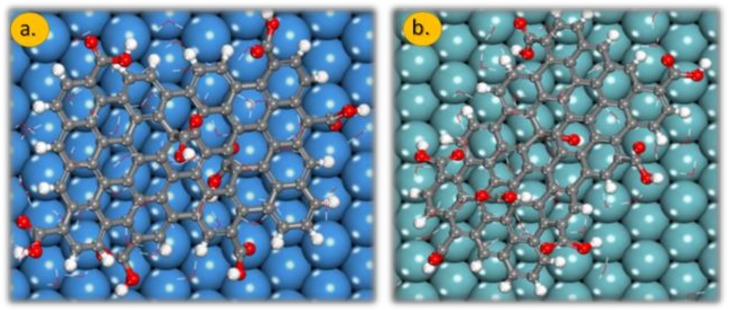
The adsorption geometry of GO-COOH onto the: (**a**) Ta (111) and (**b**) Nb (111) surface in the presence of 50 water molecules. Gray colour: C atoms, white colour: H atoms and red colour: O atoms.

The presence of water molecules drastically changes the adsorption geometry of the GO-COOH, Figure 6, top-view. In the absence of water molecules, the distance of GO-COOH (H atoms) from the surface plane of the material is around 1.8 Å; this distance increases drastically in the presence of water molecules: for the Nb (111) it is 5.7 Å, whereas for Ta (111), it is is 6.4 Å. The water molecules are intercalated between the metal (oxide) surface and GO-COOH. These molecules increase the interaction between the two partners and therefore lowers the interaction energy. These water molecules provide common solvation of both partners.

Similarly, the O atoms of the GO-COOH move further away from the material to host the intercalating water molecules. The O atoms of the side GO-COOH are closer to the surface of the metal. Energies are lower than for the metal counterpart surfaces, both in the gas phase and in the presence of water molecules. This is related to the increased GO-COOH atom distances from the oxide surfaces with regard to metal ones (Table 2).

The GO-COOH on the Ta (111)/50H_2_O is adsorbed on the side, whereas in the case of Nb (111)/50H_2_O, we observe a different adsorption geometry—the planar one. This is in good agreement with the experimental data obtained concerning the corrosion inhibition efficiency difference between Nb and Ta (the planar adsorption of GO-COOH covers a greater surface compared with the side adsorption geometry). The evaluation of the adsorption of oxides is important in the case of initial surface corrosion but also for non-uniform corrosion although we have not tested the inhibition efficiency for the Ta and Nb oxides.

### 3.2. Molecular Dynamics 

**Figure 7:**
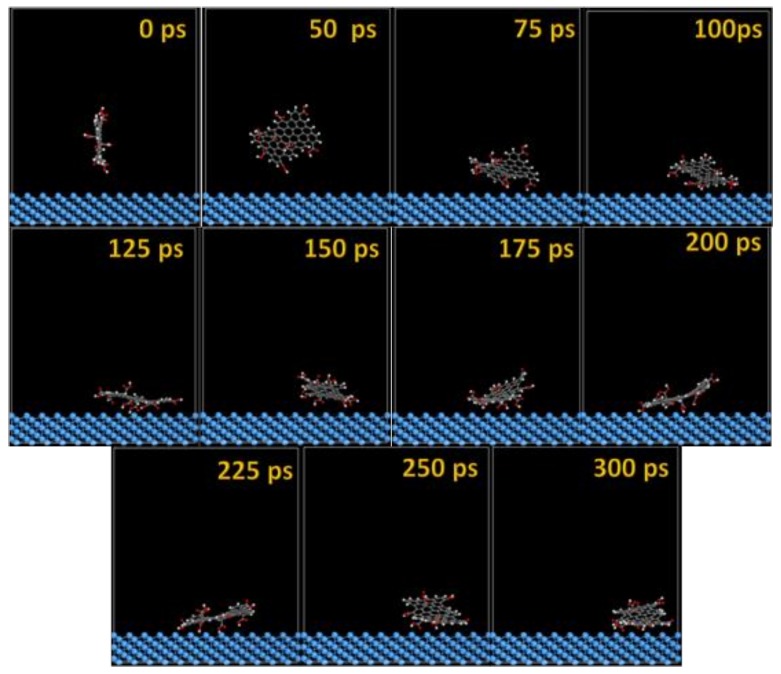
Time sequence interaction poses of the GO-COOH adsorbed on the Ta (111) surface.

**Figure 8:**
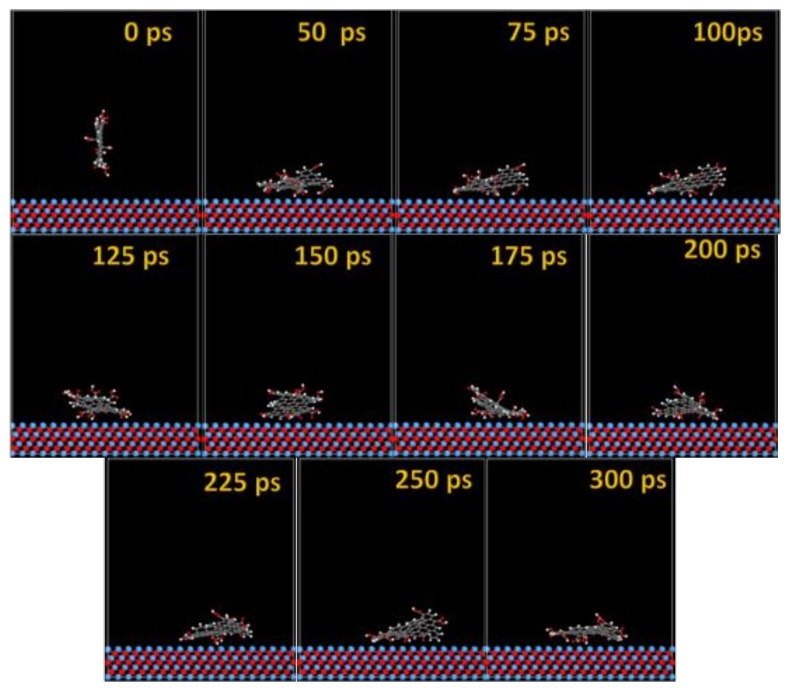
Time sequence interaction poses of the GO-COOH adsorbed on the TaO (111) surface.

The MD snapshots of GO-COOH adsorbed onto the Ta and TaO (111) surfaces with the upright direction at different simulation times are displayed in Figure 7 and Figure 8. 

After several ps of simulation time, on both of the surfaces (Ta and TaO), the GO-COOH has a tendency for surface interaction. The interaction with the TaO surface appears to progress slightly faster than with the Ta surface (GO-COOH that sits on the surface after 70 ps); in this case after 45 ps of the simulation time, GO-COOH sits flat on the surface, reaching the lowest energy conformation on surfaces takes some hundreds ps after which there is a tendency for the GO-COOH to adsorb closest to the surface. 

**Figure 9:**
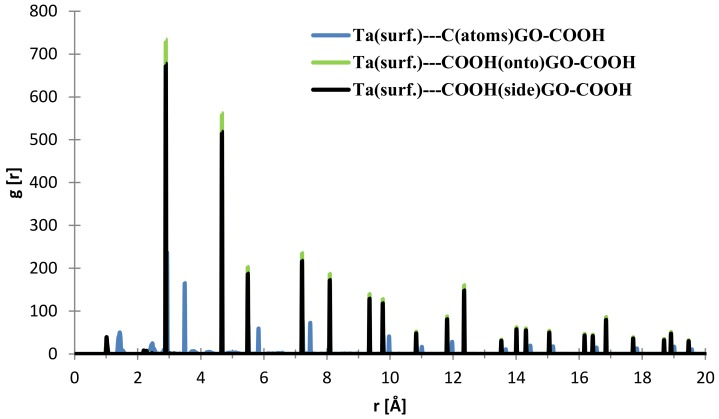
RDFs between different GO-COOH atoms: tantalum (111) surface atoms.

From Figure 9, the nearest distance distribution for most of the C (GO-COOH)–Ta (surface) atoms during the 300 ps MD simulation time has a very minor contribution at 1.03 Å, and a major one at the side –COOH group at 2.89 Å. Most of the atoms from the top COOH group during MS pass time at 2.88 Å; for the two COOH groups, there is a small RDF at a distance 2.86 Å that arises during the GO-COOH interaction with the surface in the interface vicinity.

**Figure 10:**
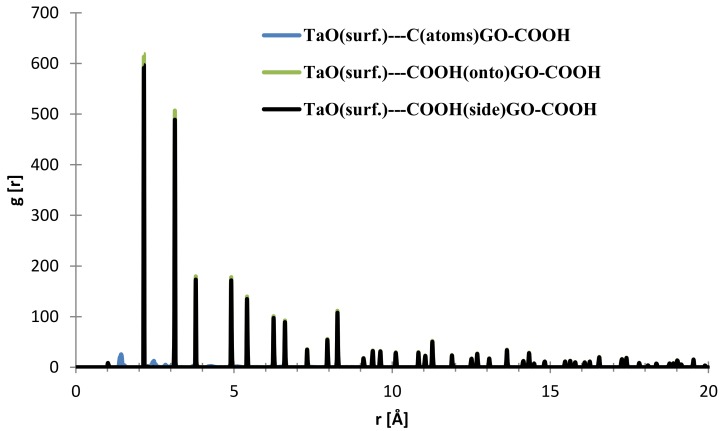
RDFs between different GO-COOH atoms: tantalum (111) surface atoms.

In the case of the interface distances for the NbO (111) surface as seen from Figure 10, the C (from GO-COOH) is positioned at 1.45 Å (small contribution) and at 2.15 Å, the top-COOH group at 1.04 Å (minor contribution), 2.16 Å and the side COOH group is positioned at 2.13 Å.

**Figure 11:**
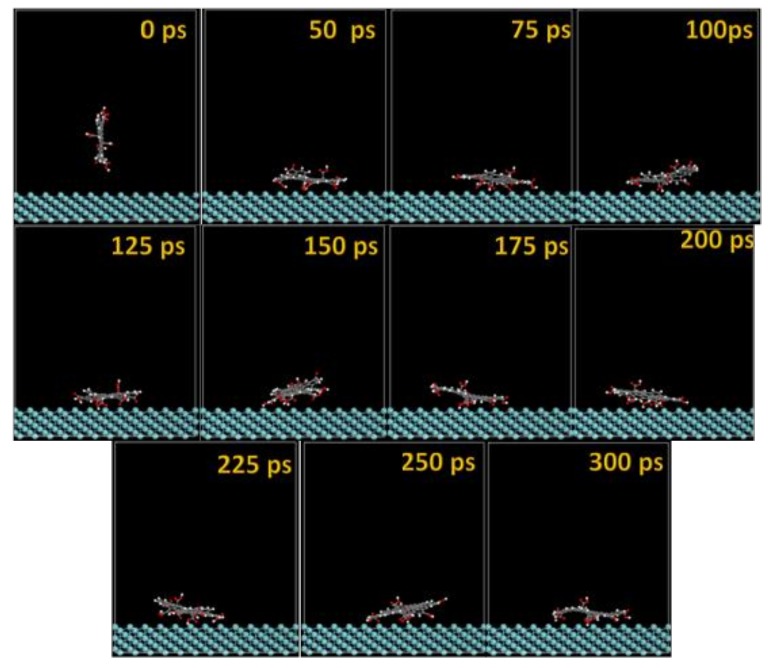
Time sequence interaction poses of the GO-COOH adsorbed on the Nb (111) surface.

**Figure 12:**
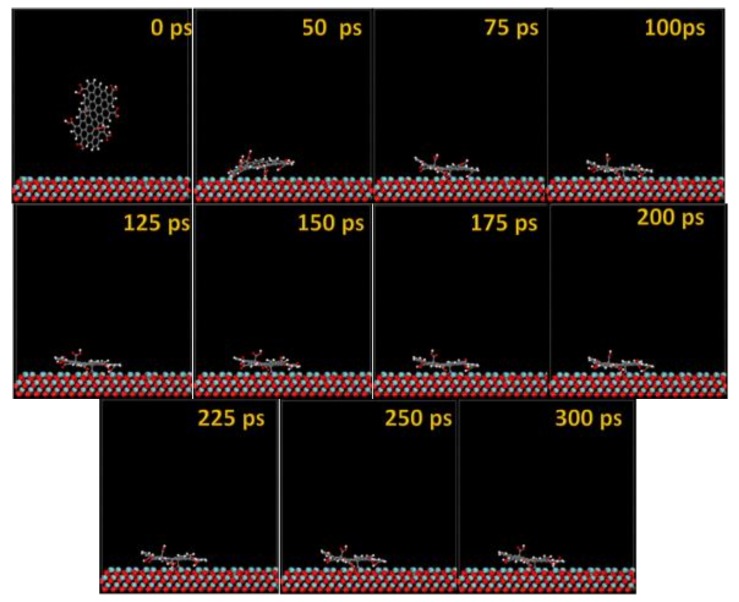
Time sequence interaction poses of the GO-COOH adsorbed on the NbO (111) surface.

The MD adsorption snapshots of GO-COOH on Nb and NbO (111) (Figure 11 and Figure 12) surfaces are similar to those of Ta and TaO.

The GO-COOH rapidly reaches the surfaces; after 45 ps, it lies flat on the surfaces and tends to find the most favorable adsorption geometry that leads to the lowest energy of the systems.

**Figure 13:**
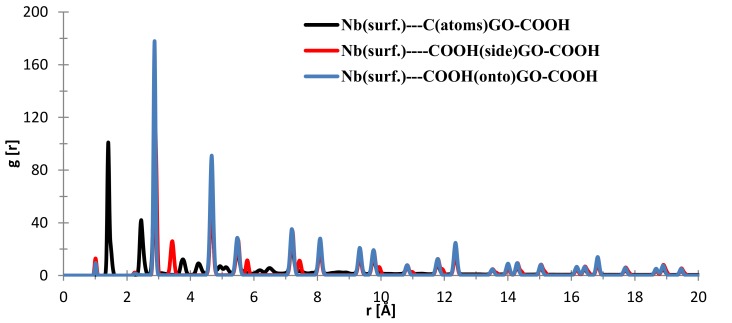
RDFs between different GO-COOH atoms: niobium (111) surface atoms.

To evaluate the interaction, RDF calculations involving the surface atoms of slab models and the GO-COOH molecule were analyzed. In Figure 13, the nearest distance distribution for most of the C (GO-COOH)–Nb (surface) atoms during the 300 ps MD simulation time is at 1.41 Å, the side –COOH group has major distance distributions at 2.84 and 3.45 Å, whereas most of the atoms from the top COOH group during MS spend time at 2.83 Å, for the two COOH groups, there is a small RDF at a very short distance of 1.1 Å that arises during the GO-COOH contact with the surface and its folding at the contact interface. The small distributions at high r values above 5 Å arise from the fact that the MD is started at a distance 20 Å from the surface. 

**Figure 14:**
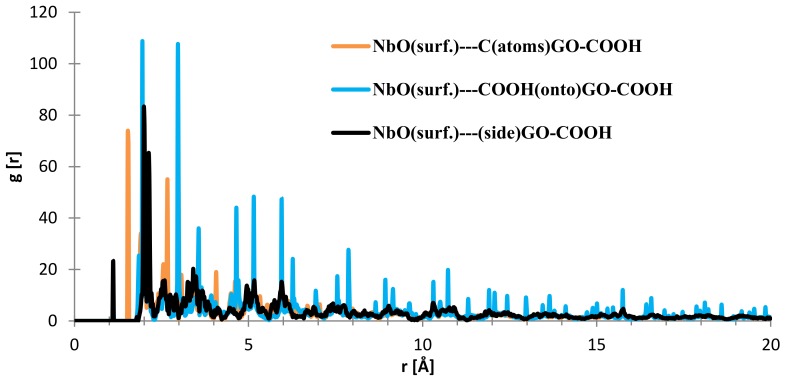
RDFs between different GO-COOH atoms: NbO (111) surface atoms.

Regarding the interface distances in the case of NbO (111), as seen from Figure 14, the C (from GO-COOH) is positioned at 1.85–1.95 Å (two RDF peaks), the side-COOH group at 1.1 Å (important contribution) and the top COOH group fluctuates at 1.94 and 2.97 (meaning that the search for the most stable position has more complex dynamics than in the case of the Nb surface; this is also evidenced by the RDF spikes with decreased intensity from 20 to 4 Å).

In general, these short distances lead to relatively strong interactions of GO-COOH with Ta, Nb, and TaO, NbO surfaces. The calculated adsorption energies for the MD systems are, as in the case of MC calculations, very similar for both of the systems. In the case of Nb (111), the adsorption energy is −0.380 kcal/mol·Å2, whereas for the Ta (111) surface, this value is −0.382 kcal/mol·Å2.

## 4. Conclusions

This work describes the corrosion inhibition of niobium and tantalum by modified graphene oxide. Experimental results from potenciodynamic measurements will be subjected to theoretical calculations.

The results of the inhibition efficiency showed that the use of GO-COOH for corrosion inhibition in the aqueous acidic media, in the case of Nb, can decrease the corrosion by up to 70%, whereas for the Ta, the inhibition efficiency reaches 60%. These data are important as a starting point to see if the MC and MD simulations will be in agreement and offer some insights regarding the molecular behavior of GO-COOH at the studied material interface. Thus, the interaction of GO-COOH on the Nb (111), NbO (111), Ta (111) and TaO (111) surfaces was investigated by MC and MD simulations with the aim to better understand the interface interaction of GO-COOH. The MC simulations are in agreement with the experimentally observed behavior of GO-COOH; the calculated interaction energy was a bit stronger between the Nb (111)/50H2O//GO-COOH than the Ta (111)/50H2O//GO-COOH. The MD and MC simulation can be helpful to design and explore GO structures prior to experimental measurements that tend to exhibit more pronounced inhibition efficiencies, leading to decreased corrosion cost. 

## Figures and Tables

**Table 1:** Results of Tafel slope analysis of the polarization plots at 298 K.

Studied System (in 0.1 M H_2_SO_4_)	B_c_(V dec^−1^)	B_a_(V dec^−1^)	j_corr_ (Acm^−2^)	Ecorr (V/SCE)	IE (%)
Nb	0.167	0.092	2.46 × 10^−6^	−0.205	-
Nb + GO-COOH	0.139	0.149	7.33 × 10^−7^	−0.175	70.22
Ta	0.165	0.131	5.71 × 10^−7^	−0.247	-
Ta + GO-COOH	0.145	0.225	2.05 × 10^−7^	−0.240	64.07

**Table 2:** Adsorption distances for GO-COOH onto Nb (111), NbO (111), Ta (111) and TaO (111) surfaces in the absence and presence of 50 water molecules.

Substrate (POS = Plane of Substrate)	d_H(GO-COOH)-POS (Å)_	d_O(GO-COOH)-POS (Å)_	d_c(GO-COOH)-POS (Å)_	d_H(GO-COOH)-POS (50H_2_O) (Å)_	d_O(GO-COOH)-POS (50H_2_O) (Å)_	d_centroid(GO-COOH)-POS (50H_2_O) (Å)_	Ads. Geometry
Nb_(gas)_ 111	1.850	1.534	2.259	-	-	4.216	Planar
Nb_(H_2_O)_ 111	5.722	6.697	6.947	1.705	2.262	8.727	Planar
NbO_(gas)_ 111	2.132	1.716	1.419	-	-	4.345	Planar
NbO_(H_2_O)_ 111	2.131	1.810	2.653	1.279	1.182	4.897	Planar
Ta_(gas)_ 111	1.818	1.533	2.285	-	-	4.342	Planar
Ta_(H_2_O)_ 111	6.419	5.689	6.621	2.419	2.651	10.607	Side
TaO_(gas)_ 111	0.841	0.758	1.261	-	-	3.305	Side
TaO_(H_2_O)_ 111	2.122	2.156	2.981	2.244	2.648	7.988	Side
